# Open Reduction and Internal Fixation Using a Posterolateral Approach for a Posterolateral Osteochondral Lesion of the Talus: A Case Report

**DOI:** 10.7759/cureus.102542

**Published:** 2026-01-29

**Authors:** Yu Kobai, Takahide Sasaki, Yukihiro Nakagawa, Hiroshi Yamada

**Affiliations:** 1 Department of Orthopaedic Surgery, Wakayama Medical University Kihoku Hospital, Katsuragicho, JPN; 2 Department of Orthopaedic Surgery, Wakayama Medical University, Katsuragicho, JPN

**Keywords:** a case report, internal fixation, osteochondral lesion of talus, posterolateral approach, surgery

## Abstract

Osteochondral lesions of the talus (OLT) are characterized by damage to the articular cartilage and subchondral bone, resulting in ankle pain. In open surgical management of OLT, selecting an appropriate approach to the talus based on lesion location is a major challenge. Posterolateral OLTs are rare, and no previous studies have reported open surgery using a posterolateral approach for these lesions. This report describes a 24-year-old man with a posterolateral OLT who underwent open reduction and fixation via a posterolateral approach and achieved a favorable outcome. This case provides clinically relevant information for considering open surgical approaches to posterolateral OLT.

## Introduction

Osteochondral lesions of the talus (OLT) are characterized by damage to the articular cartilage and subchondral bone, leading to ankle pain [1,2]. They arise after acute ankle trauma, including fractures and sprains, or through repetitive microtrauma associated with chronic ankle instability [3,2]. OLTs most frequently involve the centromedial and posteromedial talar dome, followed by the central lateral region, whereas posterolateral lesions are rare [4]. Surgical intervention is frequently indicated for symptomatic OLTs [3]. The selection of procedure is determined by lesion size, location, stability, and patient-related factors, and includes bone marrow stimulation, drilling, internal fixation, and osteochondral autograft transplantation [5-8].

Bone marrow stimulation and drilling for OLT are typically performed arthroscopically, whereas open surgery is often required for internal fixation or osteochondral autograft transplantation [9,10]. In open surgical management of OLT, selection of the surgical approach according to lesion location remains a key issue. Cadaveric studies have shown that posterolateral OLT can be accessed through a lateral approach with fibular osteotomy or through a posterolateral approach [11,12]. However, to date, no reports have described open surgery using a posterolateral approach for posterolateral OLT. This report presents a case of posterolateral OLT successfully treated with open reduction and internal fixation via a posterolateral approach.

## Case presentation

**Figure 1:**
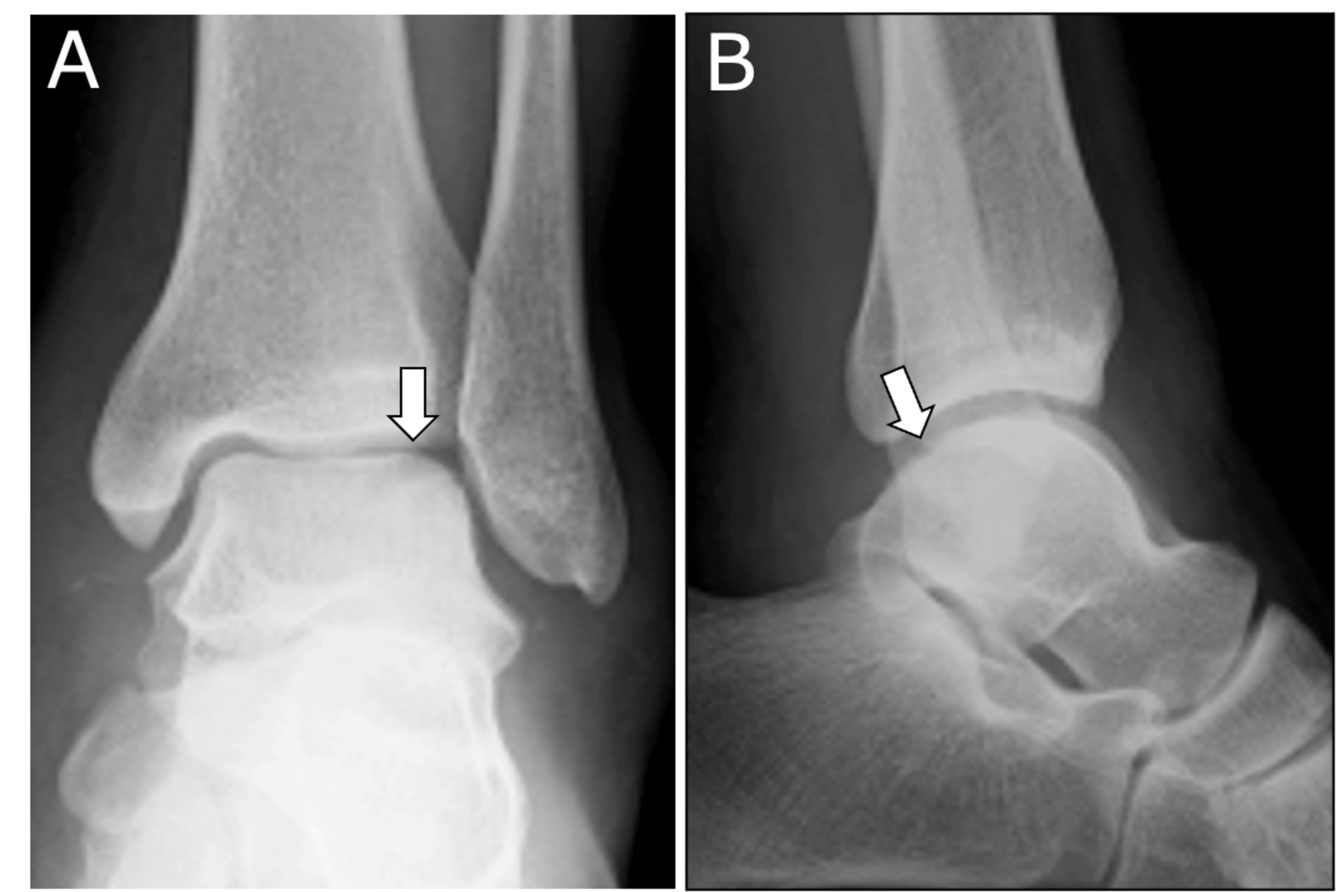
Preoperative plain radiographs A: The anteroposterior view demonstrates a radiolucent area in the lateral talar dome (arrow). B: The lateral view reveals an irregular contour of the posterior talar dome (arrow).

**Figure 2:**
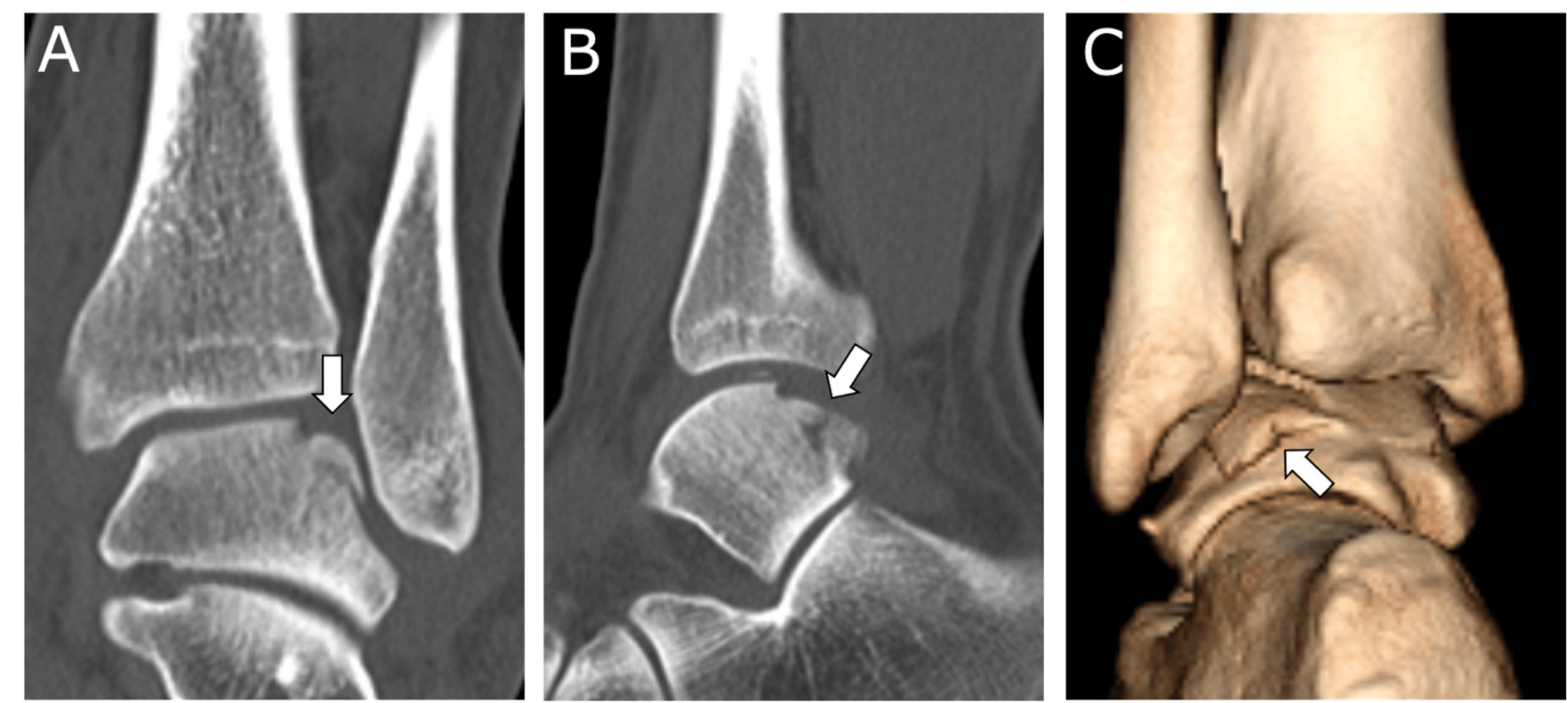
Preoperative plain computed tomography (CT) images A displaced osteochondral lesion is identified in the posterolateral talar dome (arrow). A: Coronal view; B: Sagittal view; C: Three-dimensional CT reconstruction

A 24-year-old man presented with pain and swelling of the left ankle after falling from a height of 3 m during carpentry work. He was evaluated at a nearby hospital on the day of injury and was referred to our institution the following day for definitive management. Physical examination revealed marked swelling of the left ankle without blister formation. Plain radiographs demonstrated a radiolucent area in the lateral aspect of the talar dome on the anteroposterior view and an irregular contour of the posterior talar dome on the lateral view (Figure 1). Computed tomography revealed a displaced osteochondral lesion in the posterolateral talar dome measuring 15 mm in the anteroposterior dimension and 10 mm in the mediolateral dimension (Figure 2). The lesion involved 42% of the anteroposterior length of the talar dome. No other associated injuries were identified. Based on these findings, an acute posterolateral osteochondral lesion of the talus was diagnosed. Given that the lesion was larger than 100 mm² and displaced, open reduction and internal fixation were indicated. Arthroscopic management was considered technically difficult, and open surgery was therefore selected.

**Figure 3:**
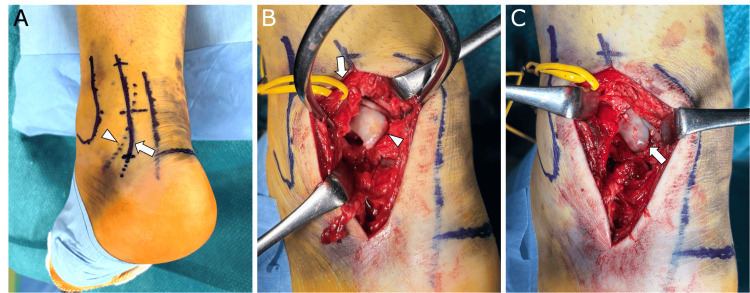
Intraoperative photographs A: The skin incision site (arrow). The sural nerve was marked under ultrasound guidance (arrowhead). B: Posterolateral approach. The sural nerve and small saphenous vein were retracted laterally (arrow). The ankle was approached between the peroneal muscles and the flexor hallucis longus muscle, and the displaced osteochondral lesion of the talus (OLT) was identified (arrowhead). C: Reduction and internal fixation of the OLT using three hydroxyapatite/poly-L-lactic acid composite implants (arrow).

Surgery was performed six days after injury, once ankle swelling had sufficiently subsided. The procedure was conducted under spinal anesthesia with the patient in the prone position, and a tourniquet was applied to the thigh. Preoperatively, the sural nerve was identified and marked using ultrasound guidance, and a 6 cm longitudinal skin incision was made at the midpoint between the posterior margin of the fibula and the lateral margin of the Achilles tendon (Figure 3A). The sural nerve and small saphenous vein were identified and retracted laterally, and the ankle was approached between the peroneal muscles and the flexor hallucis longus muscle via a posterolateral approach (Figure 3B). The posterolateral joint capsule was incised, the ankle was maximally dorsiflexed, and the displaced OLT was identified (Figure 3B). The lesion was reduced and stabilized using three hydroxyapatite/poly-L-lactic acid composite implants (Osteotrans Plus; Zimmer Biomet Holdings, Inc., Warsaw, Indiana, United States) (Figure 3C).

**Figure 4:**
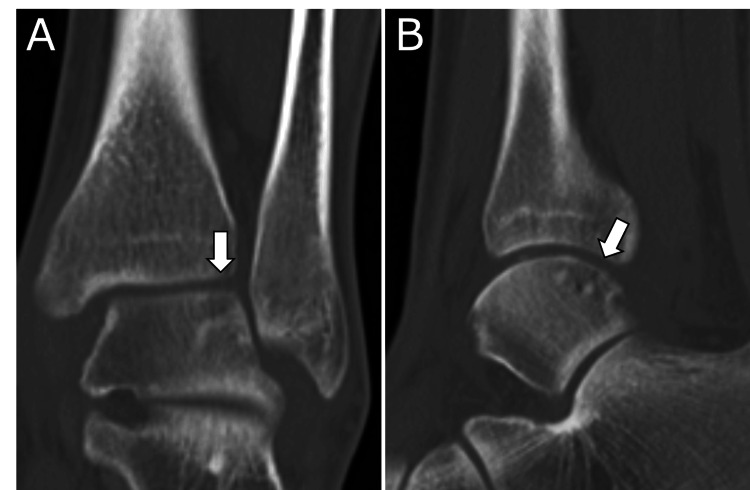
Plain computed tomography images at three months postoperatively Bone union of the osteochondral lesion is confirmed (arrow). A: Coronal view; B: Sagittal view

Postoperatively, a cast extending from the lower leg to the foot was applied for two weeks, and non-weight bearing was maintained for four weeks. Partial weight bearing with an ankle brace was initiated at postoperative week 4, and full weight bearing was permitted at postoperative week 6. Computed tomography at three months postoperatively confirmed bone union (Figure 4). At six months postoperatively, there was no pain or limitation in ankle range of motion, and the American Orthopaedic Foot and Ankle Society Ankle-Hindfoot Score was 100.

## Discussion

This report presents a case of open reduction and fixation for posterolateral OLT. The novelty of this report is that it represents the first description of open surgery using a posterolateral approach for posterolateral OLT. This case provides clinically relevant information for considering open surgical approaches to posterolateral OLT.

Posterolateral OLT is uncommon, accounting for approximately 4% of all OLT cases [4]. Only two prior surgical reports addressing posterolateral OLT have been identified [13] [14]. Sivasamy et al. described posterior ankle arthroscopy with iliac osteochondral autografting performed with the ankle in dorsiflexion [13]. Youn et al. reported open osteochondral allograft transplantation via an anterolateral approach combined with controlled burring of the anterior lip of the tibia in plantarflexion [14]. In contrast, the present case was treated with open reduction and fixation through a posterolateral approach with the ankle in dorsiflexion. Although all cases involved posterolateral OLT, the surgical techniques and approaches differed depending on whether the lesion was acute or chronic, as well as on lesion size and patient-related factors. These findings indicate that surgical strategy should be tailored to the individual pathology of each posterolateral OLT.

Several cadaveric studies have evaluated open surgical approaches for OLT [11,12,15,16]. In a systematic review of nine cadaver studies involving 83 cadavers and 113 ankles, Sripanich et al. reported that a lateral approach with fibular osteotomy permitted access to 90.9% of the anterior lateral talar dome, whereas a posterolateral approach allowed access to 37% of the posterior lateral talar dome [11]. Although the lateral approach with fibular osteotomy provides wide exposure of lateral talar lesions, it is associated with disadvantages, including fibular shortening, articular incongruity, large incisions, excessive retinacular stretching, prolonged immobilization, nonunion, malunion, and potential chondral damage [13] [15]. In the present case, the lesion involved the posterior 42% of the lateral talar dome. Even without complete access to the anterior extent of the lesion, reduction and fixation were considered achievable, and a posterolateral approach was therefore selected. 

To approach the posterolateral lesion, maximal ankle dorsiflexion was required in this case. Sivasamy et al. likewise emphasized the importance of ankle dorsiflexion in their report of posterior ankle arthroscopy for posterolateral OLT [13]. Ankle surgery is often performed under sciatic and saphenous nerve block anesthesia, which requires placement of the tourniquet on the lower leg [17,18]. This position compresses the triceps surae and restricts ankle dorsiflexion. In the present case, spinal anesthesia was used to allow tourniquet placement on the thigh.

For a posterolateral approach to posterolateral OLT, a surgical setup that allows maximal ankle dorsiflexion is essential. However, because this report describes a single case and lacks long-term follow-up beyond one year, the findings have limited generalizability. Further studies with larger cohorts and longer follow-up are required to evaluate the effectiveness of the posterolateral approach for posterolateral OLT.

## Conclusions

This report presents a case of posterolateral OLT that was successfully treated with open reduction and internal fixation using a posterolateral approach. This case provides clinically relevant information for considering open surgical approaches for posterolateral OLT. Although the posterolateral approach may represent an effective option in the surgical management of posterolateral OLT, further studies are required because this report is limited to a single case.
